# Transcriptomic dataset of cultivated (*Sesamum indicum*), wild (*S. mulayanum*), and interspecific hybrid sesame in response to induced *Macrophomina phaseolina* infection

**DOI:** 10.1016/j.dib.2020.106448

**Published:** 2020-10-21

**Authors:** Debabrata Dutta, Vivek Kumar Awon, Gaurab Gangopadhyay

**Affiliations:** Division of Plant Biology, Bose Institute (Main Campus), 93/1 APC Road, Kolkata - 700009, India

**Keywords:** Sesame, *Macrophomina* infection, Transcriptome, Dataset, SSR, SNP, Molecular marker

## Abstract

We report here the data of transcriptome sequencing of control and infected sesame genotypes. Sesame is an emerging oilseed crop [1]. The destructive soil-borne fungi *Macrophomina phaseolina* Tassi (Goid) causes charcoal rot of sesame, leading to high (>50%) yield loss. Most of the high-yielding sesame cultivars (*Sesamum indicum*) of India are susceptible to charcoal rot. Wild sesame, *Sesamum mulayanum* shows a high degree of tolerance against many pathogens [2]. We have earlier developed an interspecific hybrid between Indian cultivated sesame and *S. mulayanum*. The parents and the F_6_ recombinant constitute the three experimental genotypes in the present report. The seedlings were infected with *M. phaseolina*. The data of the infected and control (mock-inoculated) transcriptome is presented. The RNA-seq by Illumina NovaSeq 6000 technology generated 2.9 × 10^8^ paired-end reads. We deposited the data in NCBI sequence read archive (SRA) with accession number PRJNA642699. The *de novo* assembly of clean reads generated 106,295 unigenes with an average length of 1,342 bp covering 1.42 × 10^8^ nucleotides. The screening of 106,295 unigenes with MISA and SAMtools software resulted in the identification of 26,880 simple sequence repeats (SSRs), 90,181 single nucleotide polymorphisms (SNPs), and 25,063 insertion deletions (InDels). Apart from mono-base repeats, di-nucleotides repeats (42.51%) were found to be the most abundant, followed by tri-nucleotides (14.28%) among the SSRs. Subsequently, we have designed 22,494 pairs of primers based on perfect di and tri-nucleotide SSRs. Transitions (Ts, 60%) were the most abundant substitution type among the SNPs followed by transversions type (Tv, 40%), with a Ts/Tv ratio of 1.48. The development of genic-SSR markers and SNP information will pave the way for molecular marker-assisted breeding of sesame for tolerance against charcoal rot.

## Specifications Table

**Table:** 

Subject	Agricultural and Biological Sciences (General)
Specific subject area	Molecular plant pathology
Type of data	Table Chart Graph Figure
How data were acquired	Healthy sesame genotypes were infected with *Macrophomina phaseolina* (Mp). The total RNA was isolated after 72 hpi. After checking RNA quality, cDNA library was prepared and sequenced with Illumina NovaSeq 6000. Raw data were deposited in NCBI Sequence Read Archive (SRA).
Data format	Raw Analyzed
Parameters for data collection	Data was collected from control (mock-inoculation) and Mp-infected seedlings of three sesame genotypes (cultivated, wild and hybrid).
Description of data collection	The cDNA libraries of three sesame genotypes (control and infected in each case) were used for the transcriptome analysis. The assembled contigs were used for SSR, SNP and InDel discovery.
Data source location	Institute: Bose Institute Address: 93/1 APC Road, Kolkata 700009, India Latitude and longitude (and GPS coordinates) for collected samples/data: 22° 40′ 26.148′' N, 88° 26′ 43.548′' E
Data accessibility	1. With the article 2. Repository name: NCBI׳s Sequence Read Archive (SRA) database Data identification number: BioProject PRJNA642699 with accession numbers SRX8648465, SRX8648466, SRX8648467, SRX8648469, SRX8648470, and SRX8648471 Direct URL to data: https://www.ncbi.nlm.nih.gov/sra/PRJNA642699 3. Repository name: Mendeley Data Data identification number: doi:10.17632/nk27dkn5d7.1 Direct URL to data: http://dx.doi.org/10.17632/nk27dkn5d7.1

## Value of the Data

• It is the first report of *de novo* transcriptome dataset of a commonly cultivated Indian sesame (*Sesamum indicum*), wild (*S. mulayanum*) and the inter-specific hybrid in the response of Mp infection-causing charcoal rot.
• This transcriptome dataset will unravel the resistance mechanism to Mp by identifying defence-related genes and pathways involved during plant-pathogen interaction in sesame.
• Processed SSR/SNP data can be used to develop molecular markers for charcoal rot tolerance.
• The dataset will foster future molecular marker-assisted breeding of sesame.

## 1 Data Description

**Fig. 1:**
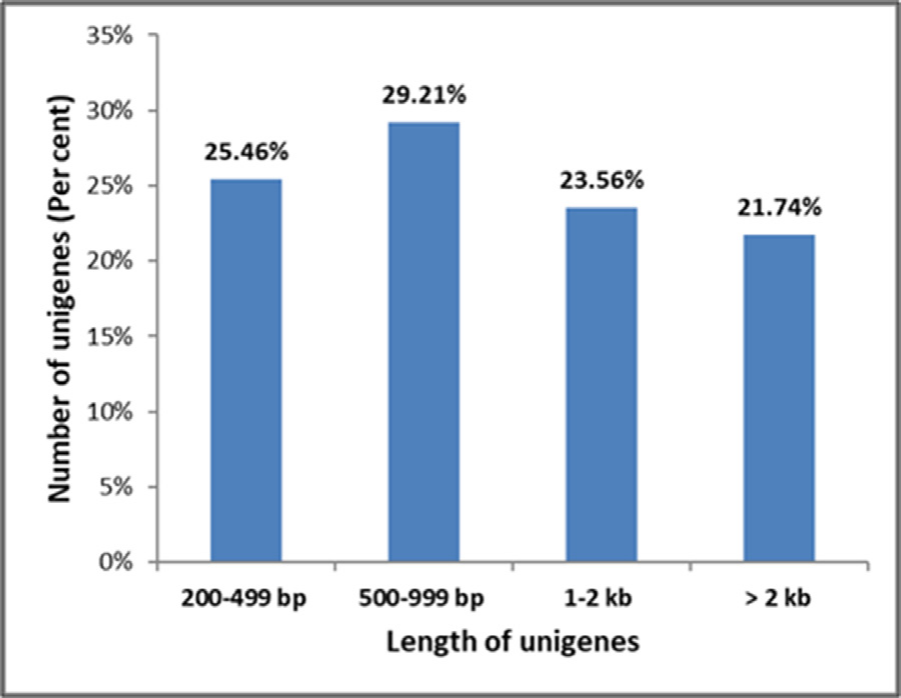
Distribution of unigenes (106,295) in different class intervals.

In the present report, we have performed transcriptome analyses using Illumina technology from leaf RNA samples of three sesame genotypes (*S. indicum, S. mulayanum*, and an interspecific hybrid, designated as recombinant throughout the manuscript) in control and Mp-infected state. This analysis generated a total of 290,670,920 raw sequencing reads from a 200 bp insert library (Table 1, Supplementary Fig. 1). Table 2 depicts the details about the quality of RNA used for library preparation. After screening the quality of data (Base quality and Phred score), the raw reads in FASTQ format were submitted to the NCBI sequence read archive (SRA) in the BioProject PRJNA642699 with the accession ID. SRX8648465, SRX8648466, SRX8648467, SRX8648469, SRX8648470, and SRX8648471 (Supplementary Table 1). A total number of 286,237,946 clean reads were generated after trimming the adaptors and removing low-quality bases. Of these reads, the *de novo* assembly by Trinity program resulted in 106,295 unigenes; with an average of 94.64% Q > 30 and 46.76% GC (Table 3). The length of the unigenes ranged from 201 to 14,433 bp with an average length of 1,342 bp. There were 27,070 (25.46 %) unigenes having a length between 200 to 499 bp, and 31,059 (29.21%) unigenes with a length between 500-999 bp. Unigenes with length more than 1000 bp and 2000 bp accounted for 25,049 (23.56%) and 23,117 (21.74%) respectively (Fig. 1, Table 3).

**Table 1:** Statistics of Illumina sequencing data; SIC, SMC and SRC represent mock-inoculated leaves and SII, SMI and SRI represent infected leaves with *Macrophomina phaseolina* (Mp) from *S.indicum, S. mulyanum* and recombinant respectively.

Sample	Raw reads	Clean reads	Clean bases (G)	Error (%)	Q20(%)	Q30(%)	GC content (%)
SIC	49,094,384	48,478,132	7.3	0.03	98.03	94.03	46.69
SII	45,191,994	44,541,856	6.7	0.02	98.27	94.69	46.52
SMC	53,117,548	52,287,126	7.8	0.02	98.31	94.77	47.21
SMI	50,934,206	50,052,396	7.5	0.02	98.3	94.74	46.93
SRC	41,320,632	40,588,856	6.1	0.02	98.37	94.93	46.96
SRI	51,012,156	50,289,580	7.5	0.02	98.32	94.7	46.27
Total	290,670,920	286,237,946	42.9	NA	NA	NA	NA

Raw Reads: The original sequencing read counts

Clean Reads: The number of reads after filtering

Clean Bases: Clean read numbers multiply read length, saved in G unit

Error Rate: Average sequencing error rate, which is calculated by Q_phred_= -10log_10_(e)

Q20: Percentage of bases whose correct base recognition rates are greater than 99% in total bases

Q30: Percentages of bases whose correct base recognition rates are greater than 99.9% in total bases

GC content: Percentages of G and C in total bases

**Table 2:** Description of the libraries for Illumina sequencing of control and Mp infected samples

Sample	Units (ng/*µ*l)	Volume(*µ*l)	Sample mass (*µ*g)	260/280	RIN value
SIC	79	17.5	1.382	2.10	6.7
SII	44	18.5	0.814	2.12	7.0
SMC	92	21.5	1.978	2.13	6.6
SMI	37	22.5	0.832	2.13	7.3
SRC	40	19.5	0.780	2.15	6.8
SRI	113	20.5	2.316	2.14	6.1

SIC: *S. indicum* control, SII: *S.indicum* infected, SMC: *S. mulyanum* control, SMI: *S. mulyanum* infected, SRC: *Sesamum* recombinant control, SRI: *Sesamum* recombinant infected

**Table 3:** Statistics of *de novo* assembly of control and Mp infected transcriptome of three sesame genotypes

Characteristic	Details
Total number of unigenes	106,295
Minimum length (bp)	201
Maximum length (bp)	14,433
Average length (bp)	1,342
Median length (bp)	880
Number of contigs 200-499 bp	27,070
Number of contigs 500-999 bp	31,059
Number of contigs 1-2 kb	25,049
Number of contigs ≥2000 bp	23,117
N50 value	2098
Average Q30(%)	94.64
Average GC content(%)	46.76

**Fig. 2:**
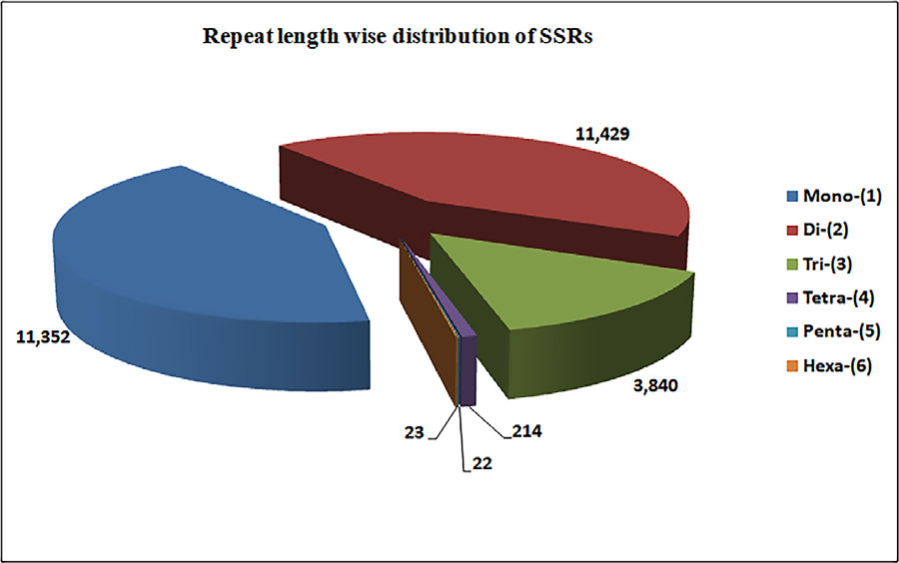
Pie chart showing the distribution of different categories of EST-SSRs (26,880) based on base pair repeats.

**Fig. 3:**
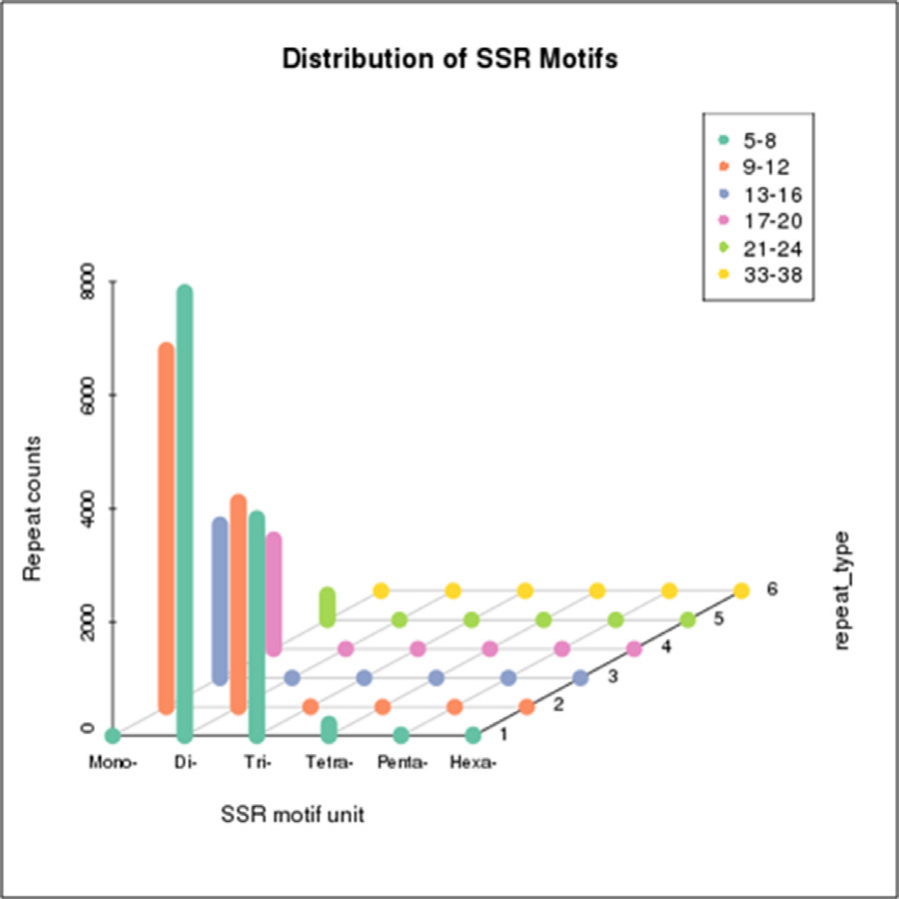
Distribution of SSR motifs. X-axis indicates type of SSR; Y-axis indicates the frequency of repeat type; Z-axis indicates number of SSR.

**Fig. 4:**
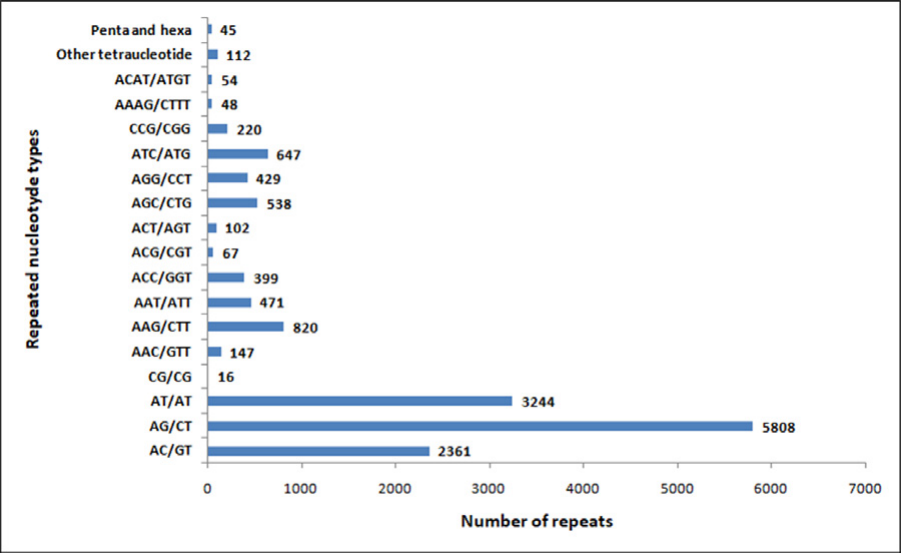
Distribution of SSR types found in the unigenes (data of mononucleotide repeats are not shown). The most predominant repeat types are the di-nucleotides (AG/CT and AT/AT).

**Fig. 5:**
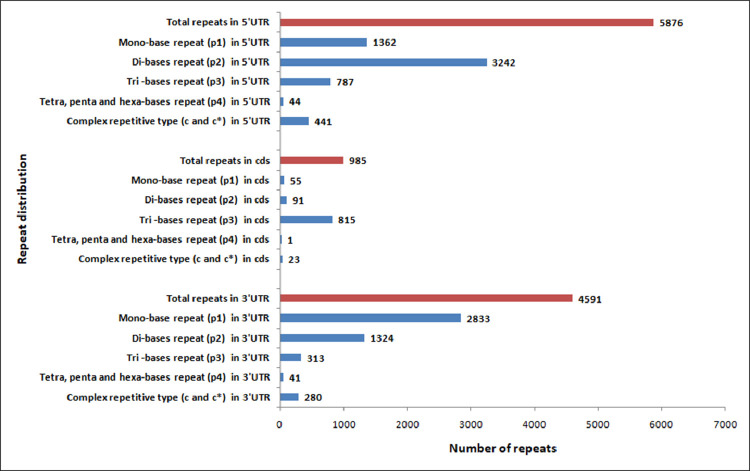
Frequency and distribution of SSRs in coding sequence and untranslated regions (UTRs). There were 5,876 (23.43%) SSRs in 5’UTR region, 985 (3.92%) SSRs in CDS and 4,591 (18.31%) SSRs in 3’UTR. Data of 13,617 (54.32%) SSRs are not shown here, as they could not be classified into any of the three classes due to lack of detection of any ORF.

**Fig. 6:**
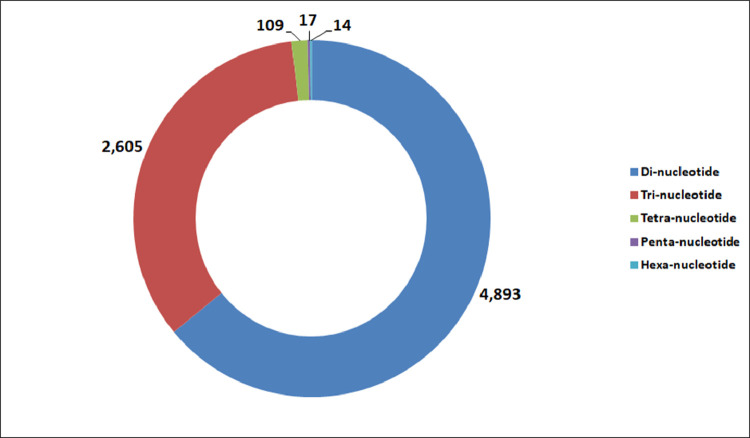
Distribution of 7,638 genic SSRs (30.46%) on which primer pairs were designed.

We identified 26,880 SSRs from 20,842 (19.6%) unigenes; with an average frequency of one SSR per 5.3kb. More than one SSR was present in 4,596 unigenes, and the number of SSRs in compound formation was 1,811 (Table 4). Apart from the mono-nucleotide repeats (11,352, 42.23%), di-nucleotide (11,429, 42.51%) and tri-nucleotide repeats (3,840, 14.28%) together constituted 56.79% of the identified SSRs (Fig. 2, Table 5). The microsatellite frequency decreased with the increase of repeat units for all the SSR types (Fig. 3). The repeat numbers ranged from 10–24 for mono-nucleotides, 6–14 for di-nucleotides, 5–13 and 38 for tri-nucleotides, 5–8 for tetra-nucleotides, 5–7 for penta-nucleotides, 5–7 and 12 for hexa-nucleotides (Supplementary Table 2). In the di-nucleotide SSR class, AG/CT nucleotides were the largest SSR motif (5,808, 21.60%), followed by AT/AT nucleotides (3,244, 12.06%) and AC/GT nucleotides (2,361, 8.78%), whereas CG/CG occurred only 16 times (0.06%) (Fig. 4, Supplementary Table 3). The identified SSRs were further classified into three classes, based on their position in the unigenes whether lying in 5′ untranslated region (UTR), coding sequence (CDS), or 3′ UTR. The analysis of sequence revealed the presence of 985 (3.92%) SSRs in the CDS and 9,118 (41.74%) SSRs in the UTR. Of the UTRs, 23.43 and 18.31% were accounted for 5′ UTR and 3′ UTR, respectively (Fig. 5, Supplementary Table 4). The di-nucleotide (3,242, 55.17%) repeats were most abundant in 5′ UTR, whereas tri-nucleotide (815, 82.74%) and mono-nucleotide (2833, 61.70%) repeats were preferentially present in the CDS and 3′ UTR region (Fig. 5, Supplementary Table 4). We were unable to classify 13,617 (54.32%) unigenes with SSR loci into any of the three classes since no ORF was found for their respective transcripts. Using Primer3 (version 2.3.5), we have successfully synthesized three primer pairs each for 7,638 (30.46%) SSRs motifs, which included 4,893 (19.51%) SSRs for di-nucleotide repeats and 2605 (10.38%) SSRs for tri-nucleotide repeats (Fig. 6, Table 6). The details of the primers sequence, expected product size and T_m_ for 7,638 genic-SSR primer pairs are provided in Supplementary Table 5.

**Table 4:** MISA (MIcroSAtellite)-based prediction of SSR result summary

Total number of assembled transcripts examined	106,295
Total size of examined sequences (bp)	142,640,365
Total number of identified SSRs	26,880
Number of SSR containing unigenes	20,842
Number of sequences containing more than 1 SSR	4,596
Number of SSRs present in compound formation	1,811

**Table 5:** Category wise distribution of predicted SSRs

SSR Type	Number	Percentage (%)
Mono-(1)	11,352	42.23
Di-(2)	11,429	42.51
Tri-(3)	3,840	14.28
Tetra-(4)	214	0.008
Penta-(5)	22	0.0008
Hexa-(6)	23	0.0008
Total	26,880	100

**Table 6:** The result summary of Primer3 (2.3.5 version)-based SSR specific primer

SSR Type	Total Number SSRs with Primer sequence
Di-nucleotide	4,893
Tri-nucleotide	2,605
Tetra-nucleotide	109
Penta-nucleotide	17
Hexa-nucleotide	14
Total	7,638

We identified 90,181 SNP loci, and the average SNP density in the whole transcriptome was 0.63/Kb. The details of SNPs, SNP-containing unigenes, position and distribution, are presented in Supplementary Table 6. The number of SNPs in each sesame genotype and infection stages varied from 40,287 to 49,929 (Table 7). The SNPs were further classified into non-coding and coding types. The minimum non-coding SNPs were detected in *S. indicum* infected sample (60.08%), while it was maximum in the control of *S. mulayanum* (62.25%). The occurrence of synonymous SNPs was higher (22.21 - 24.12%) than the non-synonymous SNPs (15.20 – 15.80%) (Table 7). Details of the non-synonymous SNPs, including the nucleotide and predicted amino acid substitutions are given in Supplementary Table 7.

**Table 7:** Predictions for Single Nucleotide Polymorphisms (SNPs)

		Coding SNP			
Sample	Non coding SNP	Synonymous	Nonsynonymous	Subtotal	Total (100%)
SIC	30,781(61.65%)	11,261(22.55%)	7,887(15.80%)	19,148(38.35%)	49,929
SII	25,963(60.08%)	10,421(24.12%)	6,827(15.80%)	17,248(39.92%)	43,211
SMC	26,112(62.25%)	9,316(22.21%)	6,519(15.54%)	15,835(37.75%)	41,947
SMI	29,185(62.16%)	10,603(22.58%)	7,161(15.25%)	17,764(37.84%)	46,949
SRC	25,022(62.11%)	8,989(22.31%)	6,276(15.58%)	15,265(37.89%)	40,287
SRI	29,597(61.39%)	11,284(23.41%)	7,327(15.20%)	18,611(38.61%)	48,208

SIC: *S. indicum* control, SII: *S. indicum* infected, SMC: *S. mulyanum* control, SMI: *S. mulyanum* infected, SRC: *Sesamum* recombinant control, SRI: *Sesamum* recombinant infected

**Fig. 7:**
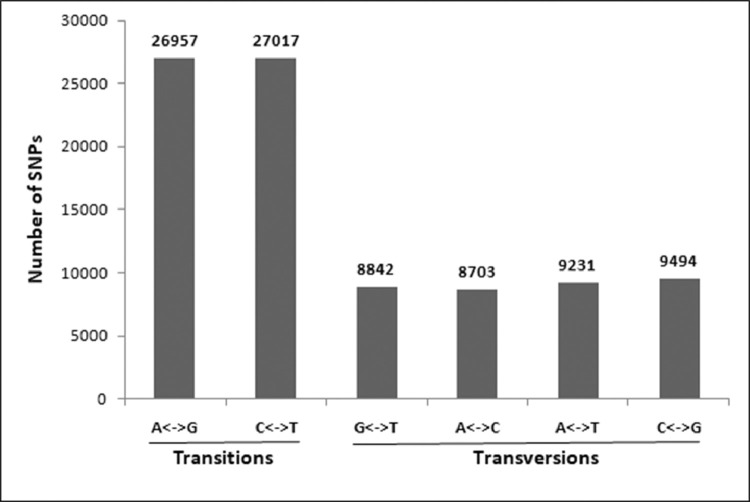
The average transitions and transversions in the SNPs (90,181).

The total number of transition (Ts) and transversion (Tv) mutations were 53,974 (60%) and 36,270 (40%) respectively, with a Ts/Tv ratio of 1.48 (Fig. 7, Table 8). Among the transition mutations, G/A and C/T showed high occurrences of 15.50 and 15.37% respectively. The C/G (5.31%) and G/C (5.20%) mutations showed higher occurrences in comparison to the other transversion mutations (Table 8). Finally, 25,063 insertions and deletions (InDels) were recorded with an average of one InDel per 5.69 kb transcriptome sequence. The details of the InDels are presented in Supplementary Table 8.

**Table 8:** The predicted mutation rate (Transition vs Transversion)

Transition type	Occurrence/Number*	Percent occurrence (%)
A->G	12,975	14.38
G->A	13,982	15.50
C->T	13,865	15.37
T->C	13,152	14.58

Transversion type		

A->C	4,263	4.72
A->T	4,560	5.05
C->G	4,796	5.31
G->T	4,573	5.07
C->A	4,440	4.92
T->A	4,671	5.17
G->C	4,698	5.20
T->G	4,269	4.73
Transition/Transversion ratio 1.48		

⁎ Out of 90,181predicted SNPs

## 2 Experimental Design, Materials and Methods

**Fig. 8:**
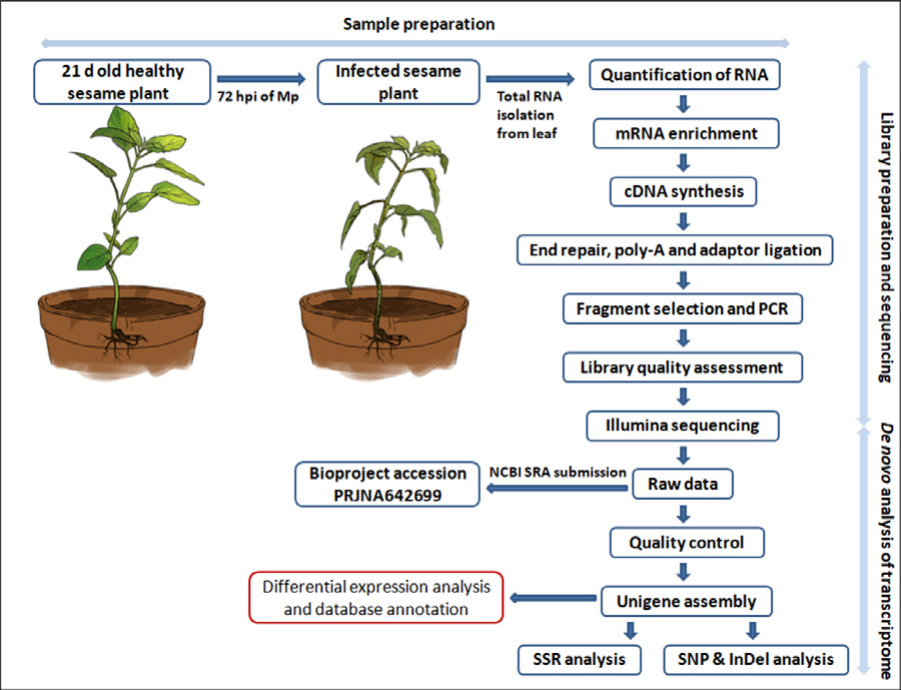
Schematic work flow used for transcriptome analysis of control and infected (Mp) sesame. Work mentioned in the red box is not shown in this paper.

The overall experimental design is depicted in Fig. 8.

### 2.1 Plant material

In this study, the parental sesame genotypes were as follows: A high-yielding cultivar of Indian sesame (*Sesamum indicum* L. - IC 131989, NBPGR germplasm collection, India), and wild sesame (*S. mulayanum* Nair). The third genotype was a recombinant (RIL) line, which we developed through interspecific hybridization. We maintain these genotypes in the experimental plots of Madhyamgram Experimental Farm (MEF), Bose Institute, Kolkata, India.

### 2.2 *In vitro* infection

A pure culture of Macrophomina phaseolina (Mp) was maintained on potato dextrose agar (PDA) plates at 30°±1°C for active growth. The inoculum was prepared by multiplication in PD broth under agitation until the development of micro-sclerotia (48 h). The micro-sclerotia were collected by filtration, rinsed and diluted with sterile distilled water. Parallel to it, the healthy seeds of three sesame genotypes were surface disinfected with 0.5% mercuric chloride for 10 min and rinsed in autoclaved double distilled water thrice. The seeds were transferred aseptically in plastic pots filled with pre-autoclaved soil-rite for germination. The germinated seedlings (21 days old) were transplanted to identical pots containing 350 micro-sclerotia g-1 soil-rite. We maintained the pots in a growth chamber for 72 h with 14 h light/10h dark cycle**.** After mock-inoculation with sterile water, the control sets were kept in the same condition [3]. The experiment was laid out as a complete randomized design (CRD). There were three replicas for both controls, and Mp inoculated genotypes, each having four plants: Three sesame genotypes × four biological replicates × two treatments (control, infected).

### 2.3 RNA isolation and library construction for sequencing

The total RNA was isolated from leaves of the control and infected plants of three genotypes using Spectrum™ Plant Total RNA kit (SIGMA). The Agilent Bioanalyzer 2100 system (Agilent Technology, USA) was used to check the RNA integrity (RIN) and quantitation using RNA Nano 6000 Assay Kit. Total RNA (1 *µ*g) was processed using NEBNext® Poly(A) mRNA Magnetic Isolation Module (NEB E7490), and six libraries were prepared with the NEBNext® Ultra™ RNA Library Prep Kit for Illumina® (NEB, USA) following the manufacturer's instruction (E7530). Index codes were added to attribute sequences to each sample. After the quality check procedures, mRNA from total RNA was enriched using oligo(dT) beads. The mRNA was then fragmented randomly in NEBNext First Strand Synthesis Reaction Buffer (5X) using divalent cations under elevated temperature. For the synthesis of the first strand of cDNA, we used M-MuLV Reverse Transcriptase (RNase-H) and random hexamer primer. It followed the generation of the second strand by nick-translation. For it we used a custom second-strand synthesis buffer (Illumina), which contained dNTPs, RNase H and DNA polymerase I. Using the exonuclease/polymerase activities, the remaining overhangs were converted into the blunt ends. For hybridization, NEBNext adaptor was ligated with the hairpin loop structure after adenylation of DNA fragments (3′ ends). The AMPure XP system (Beckman Coulter, Beverly, USA) was used for purification of the library fragments, and to select the cDNA fragments in the range of 250∼300 bp. Subsequently, 3 µl of USER Enzyme (NEB, USA) was added with the adaptor-ligated cDNA. The program of the reaction mixture was 37 °C for 15 min, followed by 5 min at 95 °C. The PCR reaction was conducted with the High-Fidelity Phusion (DNA polymerase), universal primers and Index (X) Primers for amplification of the size-selected cDNA. After purification, the PCR products were used to build the library. The quality of the library was evaluated with the Agilent Bioanalyzer 2100 system. The cBot Cluster Generation System was used to cluster the index-coded samples using PE Cluster Kit cBot-HS (Illumina). After cluster generation, six libraries were sequenced using Illumina NovaSeq 6000 to generate paired-end reads.

### 2.4 *De novo* transcriptome assembly

The raw reads that qualified Illumina's quality control were passed through in-house Perl scripts in FASTQ format. Low-quality reads containing ploy-N and adapters were removed to obtain the clean reads [4]. It followed the calculation of GC-content, sequence duplication level as well as Q20, Q30 of clean data. We deposited the raw reads in FASTQ format in the NCBI SRA database. All the downstream analyses were based on clean data with high quality. Transcriptome assembly was accomplished with clean reads using Trinity with ‘min-kmer-cov’ set to two by default and all other parameters set to default [5]. *De novo* transcriptome filtered by Corset (V 1.05) was used as a reference.

### 2.5 Discovery and analysis of SSRs

The SSRs of the transcriptome were identified and analyzed using MISA(v1.0, default parameters; minimum number of repeats of each unit size is: 1-10; 2-6; 3-5; 4-5; 5-5; 6-5, http://pgrc.ipk-gatersleben.de/misa/misa.html), and the primer sets for each SSR were designed using Primer3 (http://primer3.sourceforge.net/releases.php).

### 2.6 SNP and InDel calling

The samtools v0.1.18 and Picard - tools v1.41 were used to carry out the screening and removal of repeated reads. To conduct SNP calling and InDel calling, the GATK3 software was used [6]. The vcf files were filtered with GATK standard filter method and other parameters (cluster: 3; WindowSize:35; QD < 2.0 or FS > 60.0 or MQ < 40.0 or SOR > 4.0 or MQRankSum< -12.5 or ReadPosRankSum< -8.0 or DP < 10).

## Ethics Statement

This work did not involve any human or animal subject.

## Supplementary Materials

Supplementary material associated with this article can be found in the Mendely Data repository at http://dx.doi.org/10.17632/nk27dkn5d7.1

## CRediT Author Statement

**Debabrata Dutta:** Conceptualization, Methodology, Formal analysis, Investigation, Writing–original draft, Writing–review and editing, Visualization; **Vivek Kumar Awon:** Conceptualization, Investigation, Writing–review and editing; **Gaurab Gangopadhyay:** Conceptualization, Supervision, Writing–review and editing, Funding acquisition.

## Declaration of Competing Interest

GG is funded by an intra-mural grant of Bose Institute, Department of Science and Technology, Government of India. DD and VA are funded by the University Grants Commission of India for research fellowship. The funders had no role in the study design, data collection and analysis, decision to publish, or preparation of the manuscript. The authors declare that they have no known competing financial interests or personal relationships which have, or could be perceived to have, influenced the work reported in this article.

## Appendix Supplementary materials

Image, application 1
